# Transcriptome profiles of rice roots under simulated microgravity conditions and following gravistimulation

**DOI:** 10.3389/fpls.2023.1193042

**Published:** 2023-06-09

**Authors:** Noriyuki Kuya, Ryo Nishijima, Yuka Kitomi, Taiji Kawakatsu, Yusaku Uga

**Affiliations:** ^1^ Institute of Crop Science, National Agriculture and Food Research Organization, Tsukuba, Japan; ^2^ Institute of Agrobiological Sciences, National Agriculture and Food Research Organization, Tsukuba, Japan

**Keywords:** clinostat, gravitropism, heat shock transcription factor, rice (*Oryza sativa*. L.), RNA-Seq, simulated microgravity treatment

## Abstract

Root system architecture affects the efficient uptake of water and nutrients in plants. The root growth angle, which is a critical component in determining root system architecture, is affected by root gravitropism; however, the mechanism of root gravitropism in rice remains largely unknown. In this study, we conducted a time-course transcriptome analysis of rice roots under conditions of simulated microgravity using a three-dimensional clinostat and following gravistimulation to detect candidate genes associated with the gravitropic response. We found that *HEAT SHOCK PROTEIN* (*HSP*) genes, which are involved in the regulation of auxin transport, were preferentially up-regulated during simulated microgravity conditions and rapidly down-regulated by gravistimulation. We also found that the transcription factor *HEAT STRESS TRANSCRIPTION FACTOR* A2s (*HSFA2*s) and *HSFB2*s, showed the similar expression patterns with the *HSP*s. A co-expression network analysis and an in silico motif search within the upstream regions of the co-expressed genes revealed possible transcriptional control of *HSP*s by HSFs. Because HSFA2s are transcriptional activators, whereas HSFB2s are transcriptional repressors, the results suggest that the gene regulatory networks governed by HSFs modulate the gravitropic response through transcriptional control of *HSP*s in rice roots.

## 1 Introduction

The root is an essential organ for absorbing water and nutrients from the soil in terrestrial plants. Root system architecture is vital for the efficient acquisition of water and nutrients that are unevenly distributed throughout the ground. The root growth angle, which affects the root system architecture, is controlled by root gravitropism. *DEEPER ROOTING 1* (*DRO1*) and its homologs, positively regulate the root gravitropism and control the root growth angle in monocots (Uga et al., 2013; Ashraf et al., 2019; Kitomi et al., 2020; Feng et al., 2022; Nakano et al., 2022). A functional allele of *DRO1* causes deep-rooting and enhances the ability of drought avoidance (Uga et al., 2013). A non-functional allele of *quantitative trait locus for SOIL SURFACE ROOTING 1* (*qSOR1*) causes shallow-rooting and these roots are more effective for surface layer phosphorus absorption (Kitomi et al., 2020; Oo et al., 2021). Thus, genetic improvement of the root growth angle by manipulating root gravitropism contributes to the enhanced absorption of water and nutrients in the soil.

Gravistimulation by rotating plants 90° is a standard approach to dissecting the gravitropic response (Luschnig et al., 1998; Sedbrook et al., 1999; Boonsirichai et al., 2003; Guan et al., 2003). A three-dimensional (3D) clinostat is a device that minimizes the effects of gravity by rotating in all directions. The mounted sample is rotated three-dimensionally in two orthogonal axes. By continuously changing the law of gravity before the mounted sample is subjected to gravitational stimulation, the gravity vector is dispersed, and the effect of gravity is reduced. This condition is called “simulated microgravity” because it is imperfect. Thus, the 3D clinostat allows for simulated microgravity experiments in the laboratory on plant samples that are slow to respond to gravity (Herranz et al., 2013; **Movie S1**). Simulated microgravity treatment (SMT) using a 3D clinostat has also been used to examine the gravitropic response (Hoson et al., 1997; Herranz et al., 2013). Because all plants on the earth experience gravity, the former method captures the response to changes in the direction of gravity (dGS: directional gravistimulation). In contrast, the latter method can mimic space flight experiments and also produce the transition from simulated microgravity to forced gravity (fGS: forced gravistimulation).

The directional gravitropic mechanism of roots may be classified into four processes based on studies in the dicotyledonous model plant *Arabidopsis*: 1) graviperception in gravity-sensing columella cells (Su et al., 2017; Nakamura et al., 2019b), 2) gravity signaling following dGS mediated by LAZY1 (LZY)-like proteins (Nakamura et al., 2019a; Furutani and Morita, 2021), 3) redistribution of auxin resulting from differential polar auxin transport by *PIN-FORMED* (*PIN*) transporters (Adamowski and Friml, 2015; Han et al., 2021), and 4) differential growth in the elongation zone resulting from auxin signaling-dependent apoplast alkalinization (Barbez et al., 2017; Li et al., 2022). These processes are likely conserved in monocotyledonous plants, including rice (Uga et al., 2013; Ashraf et al., 2019; Zhang et al., 2019; Kitomi et al., 2020; Feng et al., 2022; Nakano et al., 2022). Mutations in *Defective In Outer Cell Layer Specification 1* (*DOCS1*) in rice, a leucine-rich repeat receptor-like kinase, inhibits the formation of the gravity-sensing root cap, which decreases the response to gravity (Bettembourg et al., 2017). Actin binding protein *RICE MORPHOLOGY DETERMINANT* (*RMD*) suppresses the gravitropic response of the crown root by linking actin filaments and gravitropic perception organelle amyloplasts, and *rmd* mutants show faster gravitropism (Huang et al., 2018). *DRO1* and *qSOR1* are homologs of the *Arabidopsis LZY* gene family and are involved in gravity signaling in rice (Uga et al., 2013; Kitomi et al., 2020). *OsPIN2*/*LARGE ROOT ANGLE1* (*LRA1*) plays an essential role in polar auxin transport in the rice root tip (Inahashi et al., 2018; Wang et al., 2018). The E3 ubiquitin ligase SOIL-SURFACE ROOTING 1 (SOR1) targets a noncanonical Aux/IAA protein OsIAA26 to the 26S proteasome pathway and is involved in auxin signaling in rice (Chen et al., 2018). Mutations in these genes affect the gravitropic response and alter the root growth angle; however, the gene regulatory network involved in the root gravitropic response is not well understood in rice.

In this study, we conducted a time-series RNA-seq of rice roots under simulated microgravity conditions (SMC) using a 3D clinostat and fGS to identify gravity state-responsive genes in rice roots. *Heat shock transcription factors* (*HSF*s) and *HEAT SHOCK PROTEIN*s (*HSP*s) were up-regulated by SMT and rapidly down-regulated upon release from SMT. Genes in the co-expression module, including the *HSF*s and *HSP*s share HSF-binding *cis*-elements. These results suggest that HSFs govern the gene regulatory network during the transition from simulated microgravity to forced gravity in rice roots.

## 2 Materials and methods

### 2.1 Simulated microgravity treatment using a 3D clinostat

**Figure 1:**
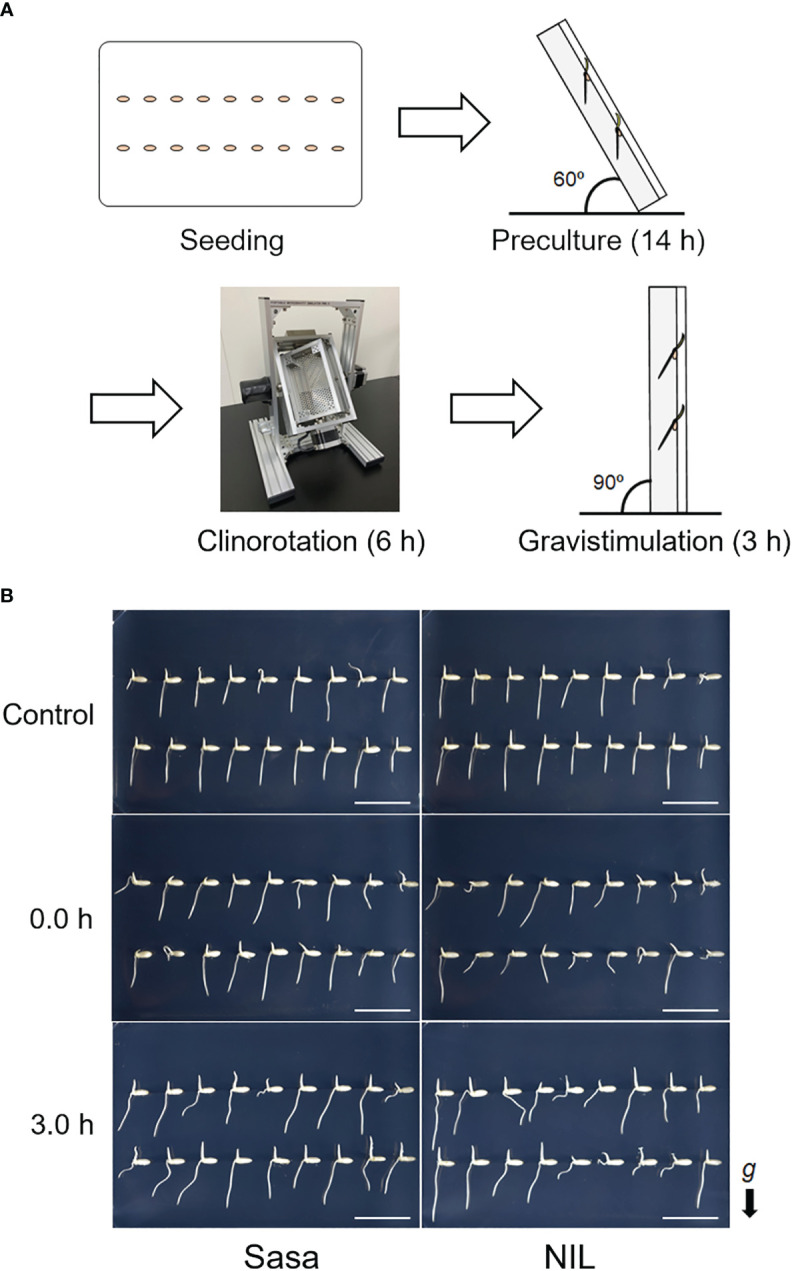
Simulated-microgravity treatment for rice roots. **(A)** Overview of simulated-microgravity treatment (SMT) and gravity treatment. Seeding: Germinated seeds were sown onto 0.4% (w/v) agarose gels in a microplate-type Petri dish. Preculture: Petri dishes were tilted 60 degrees and placed at 28°C in the dark for 14 h to allow the roots to grow down to the center of the medium. Clinorotation: The seedlings were rotated on a 3D clinostat for 6.0 h at 28°C in the dark. Gravistimulation: After SMC, gravistimulation was applied by placing the Petri dish vertically. **(B)** Representative photos after each treatment. 0.0 h and 3.0 h are different individuals. g: gravity vector, Sasa: Sasanishiki, NIL: qsor1-NIL. Bar = 2 cm.

Sasanishiki (lowland *japonica* rice) with a functional allele of *qSOR1* and a near-isogenic line with a non-functional allele of *qSOR1* (qsor1-NIL; Kitomi et al., 2020) with a Sasanishiki background was used. Hulled seeds were washed three times with sterile water. The seeds were then soaked in 1.0% (v/v) PLANT PRESERVATIVE MIXTURE (PPM™; Plant Cell Technology, Inc., USA) and incubated at 30°C to germinate for 24 hours. The germinated seeds were sown in a 0.4% (w/v) agarose gel (Sigma-Aldrich, USA) in a microplate-type Petri dish (Stem, Japan) (**Figure 1A**; Seeding). Roots protruding from the medium were susceptible to desiccation, rather than gravitropic stimulation, so the Petri dish was tilted 60 degrees and placed in the dark at 28°C for 14 h to allow the roots to elongate into the agar medium (**Figure 1A**; Preculture). They were then rotated with a 3D clinostat PMS-VI (AES, Japan) in the dark for 6.0 h at 28°C (**Figures 1A**; **S1**; **Movie S1**; Clinorotation). SMC was mimicked by adjusting the rotational speed of the 3D clinostat (X-axis 11.0 RPM/Y-axis 13.0 RPM). However, the influence of other physical stimuli that occurred by this device, such as vibrations, cannot be excluded as a possibility in this clinostat treatment. After simulated microgravity treatment (SMT), the Petri dish was placed vertically and gravistimulation was applied at 90 degrees (**Figure 1A**; Gravistimulation). Eighteen seeds were sown on each plate, and 10-16 seminal root tips with 2-3 mm were collected and pooled for RNA-seq. Seminal roots that were too short were excluded from the sampling (**Figure S2**). The root tips were collected at 0.0, 0.5, 1.5, and 3.0 h after fGS. For the control plot without SMT, the Petri dish was tilted 60 degrees for 14 h, then placed vertically, and the root tips were sampled after 6.0 h in the dark (**Figure 1B**). Therefore, the control samples are the same age as the clinorotated sample at 0h after fGS.

### 2.2 RNA extraction

Root tips were immediately frozen in liquid nitrogen and ground to a fine powder with a ShakeMaster BMS-A20TP (BMS, Japan). RNA was extracted with the RNeasy Plant Mini Kit (QIAGEN, Germany) according to the manufacturer’s instructions.

### 2.3 RNA-seq analysis

**Figure 2:**
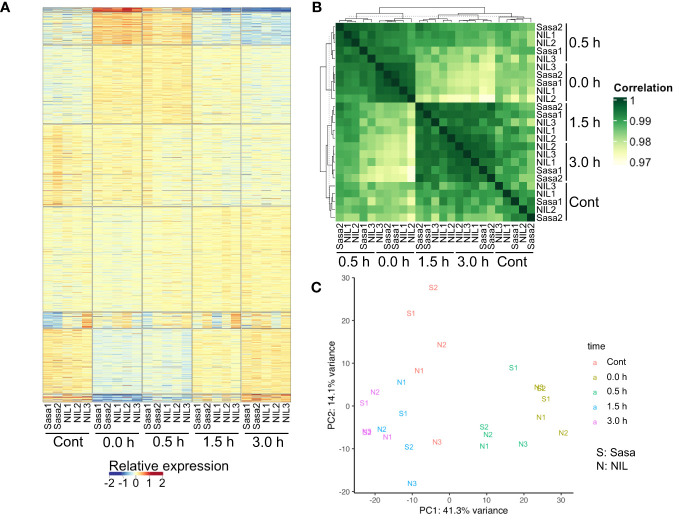
Summary of RNA-seq results. **(A)** Heatmap of gene expression levels of two and three replicates for Sasanishiki (Sasa) and qsor1-NIL (NIL), respectively. Samples were ordered by *k*-means clustering. **(B)** Heatmap of the correlation matrix of Sasanishiki and qsor1-NIL. **(C)** PCA plot based on TPM values of all expressed genes. We excluded one replicate (Sasa3) from the analysis because this replicate showed abnormal behavior (**Figure S1**).

RNA-seq libraries were prepared using the NEBNext Ultra II Directional RNA Library Prep Kit for Illumina (E7760, New England Biolabs, USA) according to the manufacturer’s instructions. Sequencing of the libraries was performed with 150 bp paired-end reads on the S4 flow cells of the Illumina NovaSeq6000 platform at Macrogen Japan. The reads were mapped to the IRGSP-1.0 genome assembly with MSU7 gene model annotation using the STAR aligner program (ver. 2.7.3a) with options “–outFilterMultimapNmax 1 –quantMode GeneCounts” (Dobin et al., 2013). Differentially expressed genes (DEGs; |log2[fold-change]| >1, the false discovery rate <0.05) were called using the glmLRT of R package edgeR (ver. 3.26.8; Robinson et al., 2010) and transcripts per kilobase million (TPM) values were computed. Genes with TPM values >2 were considered expressed. Pearson’s correlation coefficients between samples were calculated using expression levels (log2[FPKM + 1]) of all expressed genes. Relative expression in **Figures 2A**; **S3A** is shown as log2[FC to average FPKM of each gene in all samples]. Principal component analysis (PCA) was performed using TPM values of all expressed genes. All heatmaps were plotted using ComplexHeatmap (ver. 2.0.0; Gu et al., 2016). Gene ontology (GO) enrichment analysis was performed using clusterProfiler (ver 3.12.0; Yu et al., 2012) and visualized using corrplot (ver 0.84; Wei and Simko, 2017). An initial quality check using PCA and heatmap detected the wired behavior in one replicate of Sasanishiki, Sasa3 (**Figures S3A–C**). Therefore, Sasa3 was excluded from subsequent analyses, although we could not determine this reason.

### 2.4 Alignment of amino acid sequences

Clustal Omega (Sievers et al., 2011) was used to calculate the homology between HSF proteins.

### 2.5 Co-expression network analysis

Pairwise Pearson’s correlation coefficients between the genes were calculated and gene pairs with correlation coefficients >0.9 were extracted for further analysis. Co-expression modules were analyzed using the “fastgreedy.community” function of igraph (ver. 1.3.4; Csardi and Nepusz, 2006). Modules with less than five genes were discarded. *Cis*-motifs enriched in the upstream regions of genes within each co-expression module were searched using MEME (ver. 5.4.1; Bailey et al., 2015) using the option “-mod zoops -nmotifs 3 -minw 6 -maxw 13 -revcomp -markov_order 0” and the detected motifs were compared with the *Arabidopsis* DAP-seq motif dataset (Bailey et al., 2015) using TOMTOM (ver. 5.4.1; Kitomi et al., 2020) and the options “-no-ssc -verbosity 1 -min-overlap 5 -dist pearson -thresh 1e-4.”

## 3 Results

### 3.1 Root gravitropic response in Sasanishiki and qsor1-NIL

An overview of the SMT using a 3D clinostat and fGS treatment is presented in **Figure 1A**. Images of the roots at control, 0.0 h, and 3.0 h after fGS are shown in **Figure 1B**. The qsor1-NIL showed weaker gravitropism in the seminal roots compared with Sasanishiki (Kitomi et al., 2020). To identify genes downstream of *qSOR1*, we compared Sasanishiki and qsor1-NIL. Root elongation tended to be suppressed under SMC (**Figure S4A**). In the control without SMT, the root tips of both Sasanishiki and qsor1-NIL elongated toward the direction of gravity (**Figures 1B**; **S4B**). In contrast, the roots of Sasanishiki and qsor1-NIL at 0.0 h after fGS showed various directions of elongation (**Figures 1B**; **S4**). After 3.0 h of fGS, the root tips of both Sasanishiki and qsor1-NIL elongated in the direction of gravity (**Figures 1B**; **S4B**).

### 3.2 Gene expression patterns under SMC and following fGS in Sasanishiki and qsor1-NIL

To identify genes associated with the gravitropic response in rice roots, we performed a time-series RNA-seq analysis along with fGS after SMC. We considered 0.0 h after fGS as under SMC. A heatmap of 16,648 expressed genes showed that transcriptional changes mainly occurred at 0.0 h and 0.5 h after fGS in qsor1-NIL and Sasanishiki (**Figure 2A**). The correlation matrix of expression levels revealed that replicates for each condition exhibited similar patterns (*r* > 0.99; Mann-Whitney U test *P* = 3.8e-9) and the time-series gene expression profile for qsor1-NIL broadly resembled that of Sasanishiki (*r* > 0.99; Mann-Whitney U test *P* = 2.0e-10; **Figure 2B**). The controls without SMT were clustered with later-stage samples (1.5 h and 3.0 h) rather than early-stage samples (0.0 h and 0.5 h). PCA based on TPM values of the top 5% genes with the highest variability revealed that the difference in time points, rather than genotype, had more of an effect on gene expression (**Figure 2C**). This indicates that SMT-induced transcriptional changes were similar in both genotypes.

The expression of *qSOR1* tended to be slightly lower in qsor1-NIL compared with that in Sasanishiki. Although the expression levels of *qSOR1* were constant throughout the time-series in qsor1-NIL, *qSOR1* expression in Sasanishiki was down-regulated at 1.5 h after fGS (**Figure S5**). In contrast, no difference in *DRO1* expression between Sasanishiki and qsor1-NIL was observed (**Figure S5**). This suggests that the expression of *qSOR1* and *DRO1* in the root tips under SMC and subsequent fGS are similar between functional and non-functional *qSOR1* genetic backgrounds. We identified only a few DEGs between Sasanishiki and qsor1-NIL after fGS (**Figure S1D**; **Tables S1**, **S2**). The maximum number of DEGs that were up-regulated in Sasanishiki compared with qsor1-NIL was seven at 0.0 h, whereas the maximum number of DEGs that were down-regulated was 11 at 0.5 h and 1.5 h. Among these, no rice genes known to be involved in gravitropism was found. We also examined auxin-related genes associated with gravitropism; however, the expression patterns of these genes were almost identical between Sasanishiki and qsor1-NI (**Figure S6**). These results indicate that *qSOR1* is not involved in the regulation of gene expression patterns in the root tip during SMC and subsequent fGS.

### 3.3 Differentially expressed genes during fGS compared to under SMC

**Figure 3:**
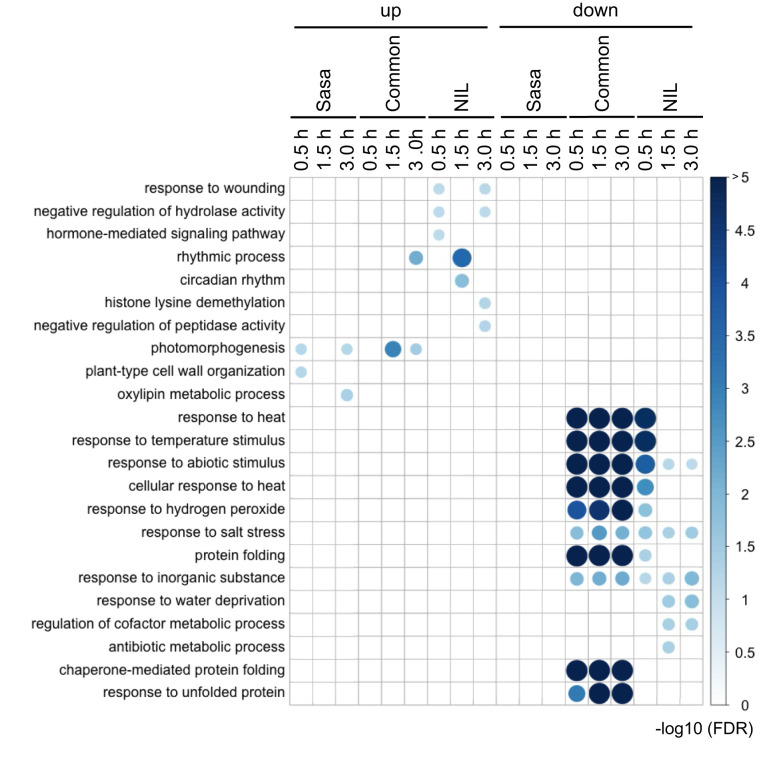
Gene ontologies enriched in each DEG cluster. The numbers on the right side of the figure indicate an adjusted p-value of -log10 at false discovery rate (FDR). The size and the intensity of the color for the circle represent the degree of the FDR value. Sasa: Sasanishiki, NIL: qsor1-NIL, common: common DEGs between Sasanishiki and qsor1-NIL.

We identified 152 and 117 DEGs in which expression was up-regulated during fGS in Sasanishiki and qsor1-NIL, respectively (**Table S3**). In addition, we identified 286 and 333 DEGs in which expression was down-regulated during fGS in Sasanishiki and qsor1-NIL, respectively (**Table S4**). GO enrichment analysis revealed that the down-regulated genes that were common to both genotypes were associated with response to heat, response to temperature stimulus, response to abiotic stimulus, cellular response to heat, response to hydrogen peroxide, protein folding, chaperone-mediated protein folding, and response to unfolded protein (**Figure 3**). We found that the three GOs (response to heat, response to temperature stimulus, and response to abiotic stimulus) were detected in qsor1-NIL, but not in Sasanishiki, at 0.5 h after fGS. Because the expression levels of the six genes in these GOs were almost the same between the two genotypes, we concluded that this discrimination was dependent upon less statistical power in Sasanishiki at 0.5 h after fGS because of the number of replicates (**Figure S7**). Based on these results, we focused our analysis on the DEGs that were common to both genotypes.

**Figure 4:**
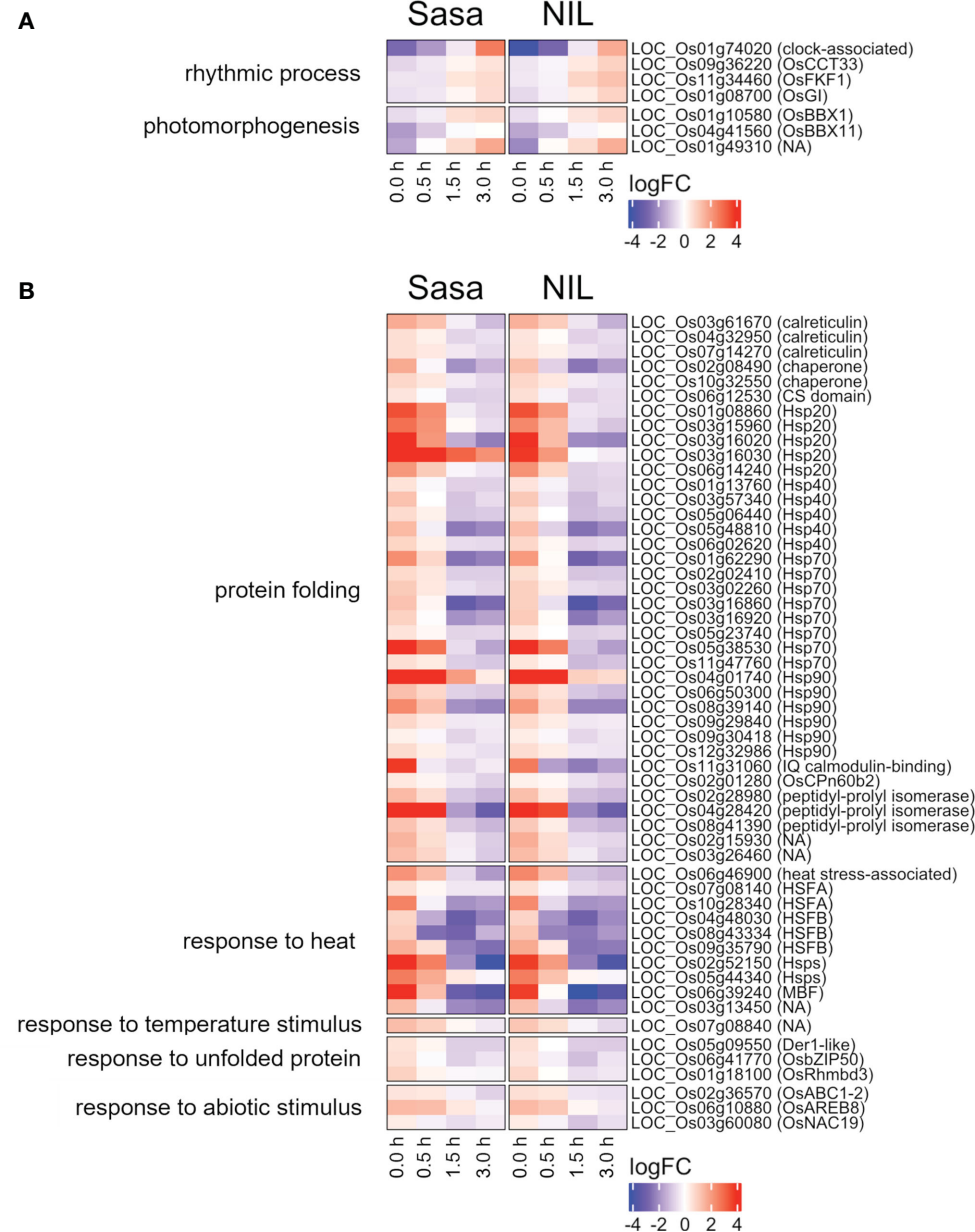
Common DEGs between Sasanishiki and qsor1-NIL in representative GOs. **(A)** Genes up-regulated during fGS. **(B)** Genes down-regulated during fGS. Gene expression is shown as logFC relative to each control. Sasa: Sasanishiki, NIL: qsor1-NIL.

Common DEGs between Sasanishiki and qsor1-NIL in representative GOs were investigated (**Figure 4**). One of the most prominent GO-enriched DEGs was protein folding. In *Arabidopsis*, HEAT SHOCK PROTEINs (HSPs) are involved in gravitropism through the regulation of auxin transport. *HSP* genes were enriched in the down-regulated DEGs (**Figure 4B**), which included five *HSP20*s, five *HSP40*s, eight *HSP70*s, and six *HSP90*s. The expression of most *HSP*s was higher at 0.0 h after fGS, but lower at 1.5 h and 3.0 h after fGS compared with those of the control without SMT (**Figure 4B**). These results indicate that the expression of *HSP* genes was induced by SMT, but repressed by prolonged fGS, likely reflecting an adaptive gravitropic response.

### 3.4 Differentially expressed transcription factor genes during SMT and fGS

**Figure 5:**
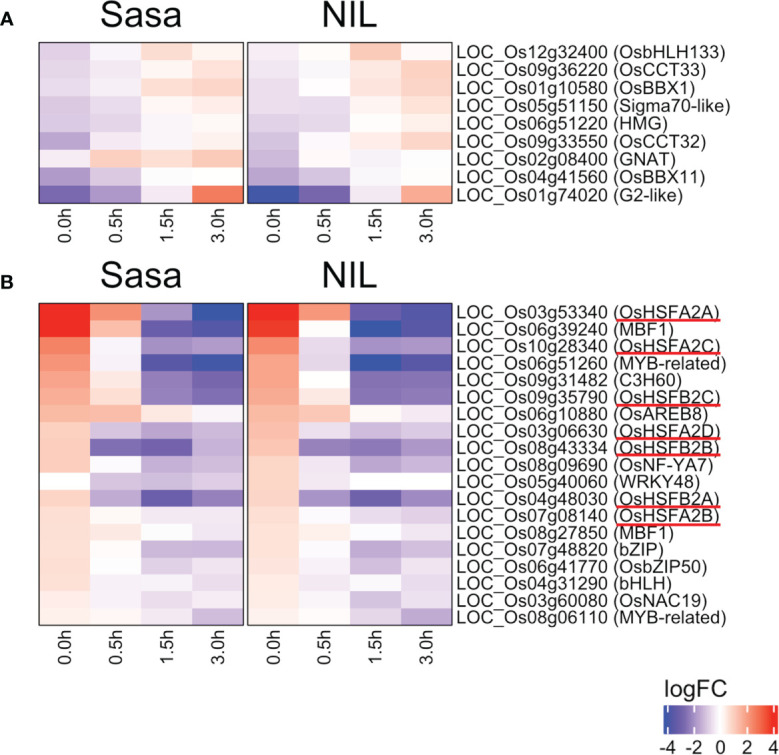
Changes in transcription factor expression during fGS. **(A)** Transcription factors in the DEGs whose expression was up-regulated during fGS. **(B)** Transcription factors in the DEGs whose expression was down-regulated during fGS. Gene expression is shown as logFC relative to each control. Sasa: Sasanishiki, NIL: qsor1-NIL. *HSF*s were underlined in red.

**Figure 6:**
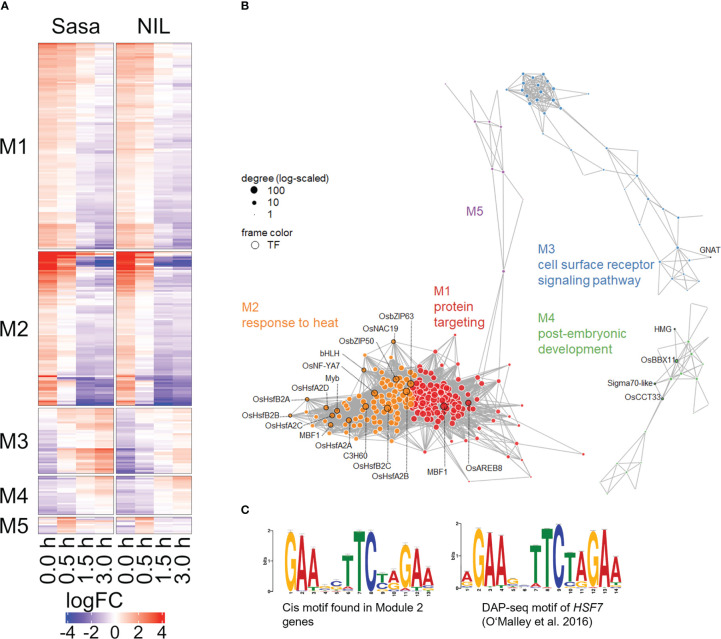
Co-expression network model of simulated-microgravity induced DEGs. **(A)** Heatmap of relative expression levels of DEGs commonly detected in Sasanishiki and qsor1-NIL. Genes were clustered by fast greedy, based on logFC relative to each control. **(B)** Five major modules (M1 to M5) presented in different colors together with the representative GO term. Transcription factors are framed in black and labeled with their gene symbols. **(C)** A representative motif found in the 200-bp upstream sequences of M2 genes (left; MEME program) and the corresponding DAP-seq result (right; TOMTOM program; O’Malley et al., 2016).

Transcription factors (TFs) regulate the expression of downstream genes and often play an important role in morphogenesis. Nine TFs were commonly up-regulated and 19 TFs were down-regulated during fGS in Sasanishiki and qsor1-NIL (**Figure 5**). Of the 19 TFs, seven were HSFs, including *HEAT SHOCK TRANSCRIPTION FACTOR A2D* (*HSFA2D*), which acts upstream of *LAZY1* and is involved in shoot gravitropism in rice (Zhang et al., 2018) (**Figure 5B**). The expression of these *HSF*s was higher at 0.0 h after fGS compared with those in the control without SMT, indicating that the expression of these *HSF*s was induced by SMT (**Figure 6**). These *HSF*s belong to the HSFA2 and HSFB2 sub-groups. *HSF*s in other sub-groups were not induced by SMT, suggesting that *HSF*s in sub-groups HSFA2 and HSFB2 are specifically involved in the forced gravitropic response in rice (**Figure S8**; **Table S5**).

### 3.5 Similarity of detected DEGs between 3D clinostat treatment and space flight

The 3D clinostat is designed to minimize the effect of gravity by randomly and continuously changing the direction of gravity; however, the extent by which SMT reproduces real microgravity in space is not guaranteed. Transcriptome analysis of plants under microgravity in space have been conducted (Zupanska et al., 2013; Vandenbrink et al., 2019; Kruse et al., 2020; Barker et al., 2023). We compared genes detected as DEGs between our SMT experiment in rice and the previous space experiment in *Arabidopsis* (Zupanska et al., 2013). Many *HSF*s and *HSP*s were commonly detected DEGs in the two studies (**Table S6**) as well as other studies (Correll et al., 2013; Choi et al., 2019; Barker et al., 2023; Paul et al., 2005; Paul et al., 2012; Kwon et al., 2015; Li et al., 2017). These results support that our SMT treatment properly mimics real microgravity in space.

### 3.6 Co-expression analysis of genes detected as DEGs after fGS

To infer the gene regulatory networks under SMCs and following fGS, we performed a co-expression analysis using DEGs commonly expressed in Sasanishiki and qsor1-NIL. The DEGs were divided into five co-expression modules (**Figures 6A, B**; **Table S7**). The seven *HSFs* that were altered under the SMC were included in module 2 (M2), which was enriched with genes associated with “response to heat.” *HSP40* genes (*LOC_Os01g13760*, *LOC_Os03g57340, LOC_Os05g48810, LOC_Os06g02620*), *HSP70* genes (*LOC_Os01g62290*, *LOC_Os03g16860*, *LOC_Os03g16920*, *LOC_Os05g38530*), *and HSP90* genes (*LOC_Os04g01740*, *LOC_Os08g39140*) were also included in M2, suggesting a possible interaction between *HSFs* and *HSP*s. We identified the enriched motif within a 200 bp upstream region of the genes belonging to M2 (55 out of 92 genes, 59.8%), which closely resembles the AtHSFA7 binding motif in the *Arabidopsis* cistrome dataset (*q*-value = 4.29e-9; **Figure 6C**) (O’Malley et al., 2016), whereas TCP motifs (M1, q = 8.21e-6) and MYB-related motifs (M4, q = 2.45e-4) were identified in the other modules (**Figure S9**, *q* < 1e-4). Because *Arabidopsis* HSF-binding motifs are nearly identical to one another irrespective of class or sub-group, we anticipate that rice HSFs also recognize similar sequences (**Figure S10**). These results suggest that HSFA2s and HSFB2 directly regulate the expression of genes in the M2 module and shape the M2 module.

## 4 Discussion

Gravitropism is a key determinant of root system architecture. We performed a time-series RNA-seq study under SMCs and following fGS, which revealed dynamic transcriptional changes and identified genes involved in the gravitropic response in rice roots. A co-expression network analysis revealed that the HSF-HSP pathway may contribute to the gravitropic response in rice roots. HSFA2D is a positive regulator of gravitropism and acts upstream of asymmetric auxin distribution in rice shoots (Zhang et al., 2018). The dGS induces the expression of *HSFA2D* and HSFA2D activates the rice LZY1 homolog *LAZY1* (*LA1*), which is involved in gravity signaling and asymmetric distribution of auxin in shoots (Zhang et al., 2018). HSP40s and HSP90s are involved in auxin perception and transport (Harrison and Masson, 2008; Wang et al., 2016). Therefore, the HSF-HSP pathway may be responsible for the asymmetric auxin distribution followed by differential growth during the gravitropic response in roots as in shoots.

Sasanishiki was more responsive to fGS compared with qsor1-NIL with respect to root growth; however, we did not observe clear differences in the expression of genes associated with root growth during SMT and following fGS between Sasanishiki and qsor1-NIL. This indicates that *qSOR1* acts independently of transcriptional control. *AtLZYs*, *Arabidopsis* homologs of *qSOR1* and *DRO1*, are expressed in columella cells and AtLZYs recruit RLD proteins to the plasma membrane in the direction of gravity, which results in polar localization of the auxin efflux carrier PIN3. Thus, qSOR1 may be involved in the regulation of auxin transport or gravity signaling, in a similar manner. Interestingly, AtLZYs also interact with HSP70s and its rice homologs were up-regulated by SMT and repressed following fGS (Furutani et al., 2020). Whether HSP70 is involved in auxin transport, however, is unknown, although HSP40s and HSP90s are involved in auxin transport. HSP40s regulate the localization of PIN3 (Harrison and Masson, 2008). HSP90s stabilize PIN1 and auxin receptor TIR1 with the co-chaperones, HOPs and SGT1b (**Figure S11**; Samakovli et al., 2021; Muñoz et al., 2022). All differentially expressed *HSP40*s, *HSP90*s, *HOP*s, and *SGT1b* were assigned to the closely related modules M1 and M2 (**Figure 6B**), which indicates that these genes are tightly co-expressed under SMC and following fGS. Because HSP70s interact with co-chaperone HSP40s and cooperate with protein folding with HSP90s, HSP70s may also be involved in auxin transport. These suggest a possible crosstalk between LZYs, including qSOR1 and the HSF-HSP pathway in regulating the localization and stabilization of PINs and auxin transport. *qSOR1* is negatively regulated by auxin signaling and *qSOR1* was transiently repressed by fGS (**Figure S5**; Kitomi et al., 2020). This suggests the existence of a negative feedback loop between qSOR1-mediated auxin transport and auxin signaling-dependent repression of *qSOR1*.

Rice *LA1*, a homolog of *qSOR1* and *DRO1*, is required for gravitropism in shoots, but not in roots. HSFA2D acts upstream of *LA1* and regulates gravitropism in shoots. Seven *HSF*s, including *HSFA2D*, were up-regulated by SMT and down-regulated by fGS (**Figure 5B**). Five of these *HSF*s are responsive to gravity changes in shoots (Zhang et al., 2018, **Figure S12**), suggesting that transcriptional regulation during the gravitropic response of rice shoots and roots is similar. The expression of *LA1* is reduced in *hsfa2d*, suggesting that HSFA2D is a positive regulator of *LA1*. Whether *qSOR1* is positively regulated by *HSF*s, including *HSFA2D*, in roots is unknown; however, the expression of *qSOR1* was not increased by SMT, although the expression of *HSF*s was increased. This is the same situation that was observed in shoots, in which *LA1* was not induced by dGS when *HSF*s were up-regulated (Zhang et al., 2018; **Figure S13**). It is possible that the transcriptional regulation of *LZY*s by HSFs is indirect or requires co-factors. HSF TFs include both transcriptional activators and repressors. Members of the HSFA subfamily are activators, whereas members of the HSFB subfamily are either activators or repressors. Of the seven *HSF*s induced by SMT and repressed by fGS, three OsHSFB2s are predicted to function as repressors based on the presence of a repression domain (Lavania et al., 2018). In *Arabidopsis*, HsfB1/B2b represses the expression of *HSP*s, *HsfA2*, and *HsfB1/B2b* themselves under normal conditions (Ikeda et al., 2011). Under heat stress conditions, HsfA1s are rapidly activated by translocation from the cytoplasm to the nucleus and induce the expression of *HsfA2* and *HsfB1/B2b*. Activated HsfA2 induces *HSP*s to acquire thermotolerance; however, HsfB1/B2b represses the expression of *HsfA2* and *HsfB1/B2b*, attenuating the heat stress response to normal conditions (Ikeda et al., 2011). We hypothesize that a similar scenario may control the adaptive gravitropic response in rice roots. OsHSFs other than OsHSFA2s and OsHSFB2s, whose expression is constant, may be activated by translocation during perturbed gravity and induce the expression of *OsHSFA2*s and *OsHSFB2*s. Activated OsHSFA2s induce the expression of *HSP*s and *OsHSFB*s for auxin redistribution and its subsequent attenuation. At this point, OsHSFBs may be inactivated by unknown mechanisms. During fGS, OsHSFBs may be promptly activated and repress the expression of *OsHSFA2*s and *HSP*s, resulting in the attenuation of the adaptive gravitropic response. However, further studies are needed to clarify the mechanism of gravitropism in rice root and utilize the knowledges to improve the root traits of monocotyledonous plants.

## Data availability statement

We have released our RNA-seq data of accession number DRA013338 on the DDBJ repository (https://ddbj.nig.ac.jp/search).

## Author contributions

YU conceived and supervised the research. YU and YK designed the research, NK and YK performed the experiments. NK, RN and TK analyzed the transcriptome data. NK, RN, TK, and YU wrote the manuscript with contributions from all authors. All authors contributed to the article and approved the submitted version.
