# Supplementary material for: Transcriptome profiles of rice roots under simulated microgravity conditions and following gravistimulation
Source: Front Plant Sci. 2023 Jun 9;14:1193042. doi: 10.3389/fpls.2023.1193042 (PMC10288856; doi:10.3389/fpls.2023.1193042)
Supplement: Supplementary file 1 [file DataSheet_1.pdf]

Supplementary Material

Transcriptome profiles of rice roots under simulated microgravity conditions and following gravistimulation

Noriyuki Kuya, Ryo Nishijima, Yuka Kitomi, Taiji Kawakatsu*, Yusaku Uga*

***Corresponding authors:**

Yusaku Uga: Yusaku Uga: yuga@affrc.go.jp

Taiji Kawakatsu: riverwin@affrc.go.jp

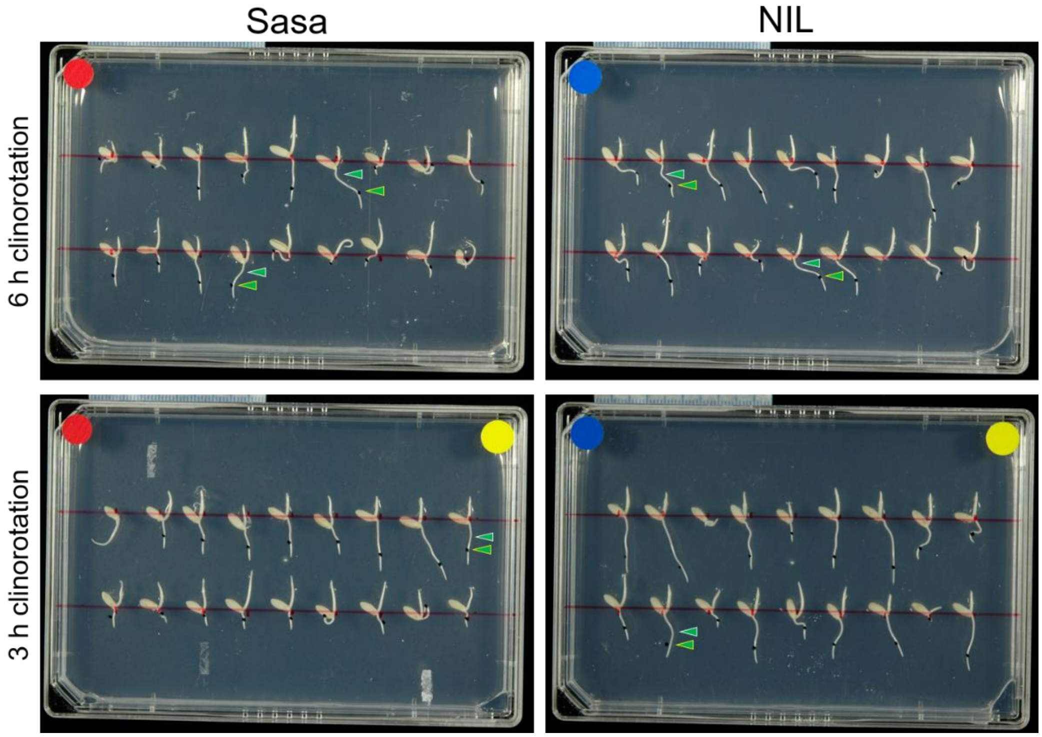

Supplementary Figure 1. Effect of clinostat treatment time.

Roots elongated in more random directions by 6 hours of clinorotation compared to 3 hours. The upper and lower green arrowheads near the root indicate root tip positions before and end of clinorotation, respectively. Sasa: Sasanishiki, NIL: qsor1-NIL.

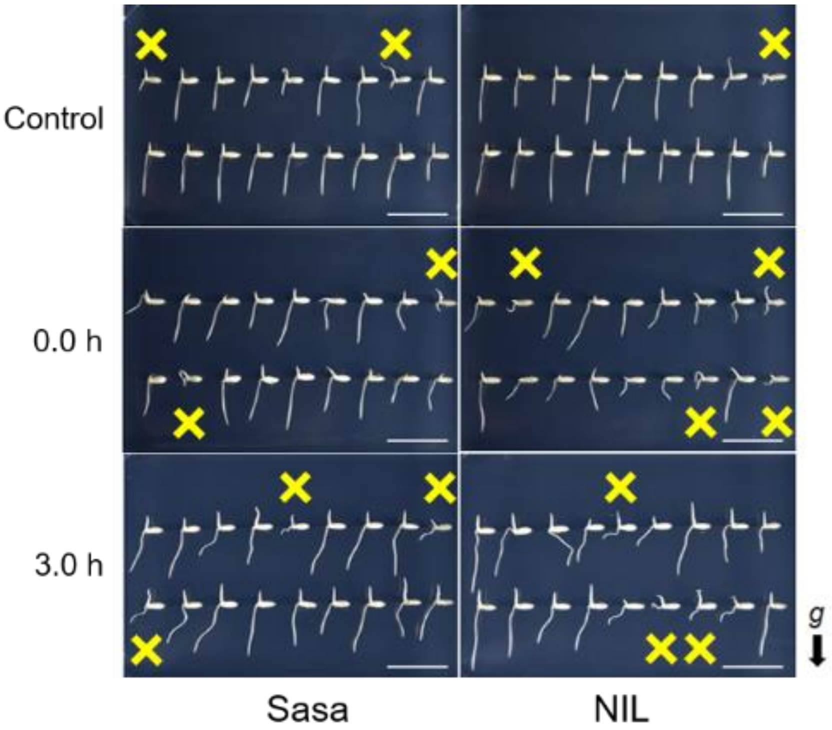

Supplementary Figure 2. Selection of samples for RNA-seq.

Seminal roots that were too short in the same treated lines were excluded from sampling. Sasa: Sasanishiki, NIL: qsor1-NIL. ×: Excluded individuals.

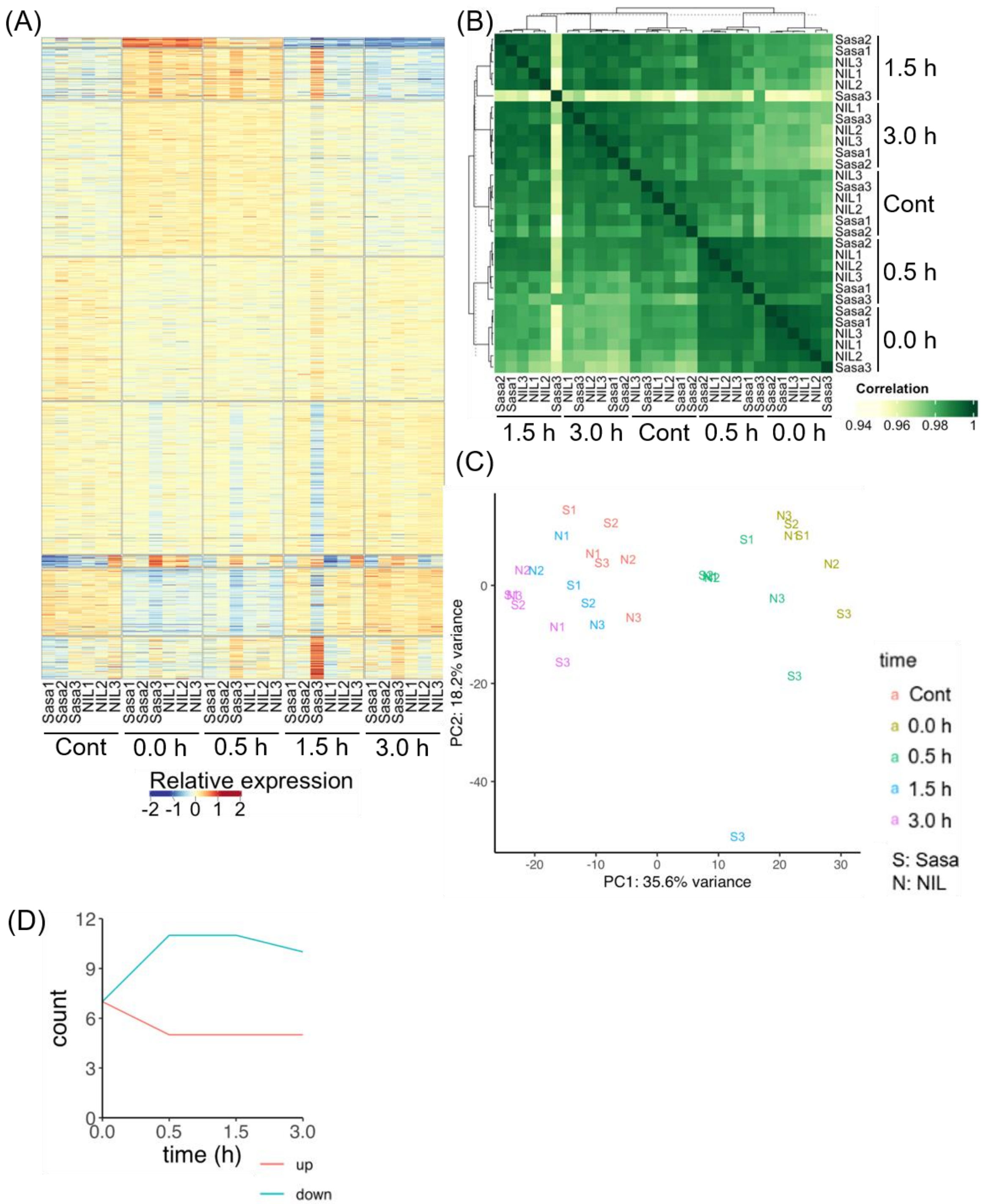

Supplementary Figure 3. Summary of the RNA-seq results.

(**A**) Heatmap of gene expression levels of three replicates for Sasanishiki and qsor1-NIL (Sasa and NIL), respectively. Samples were ordered by *k*-means clustering. (**B**) Heatmap of the correlation matrix of Sasanishiki and qsor1-NIL. (**C**) PCA plot based on TPM values of all expressed genes. (**D**) Number of DEGs up-regulated (red) and down-regulated (blue) in Sasanishiki compared with qsor1-NIL during fGS.

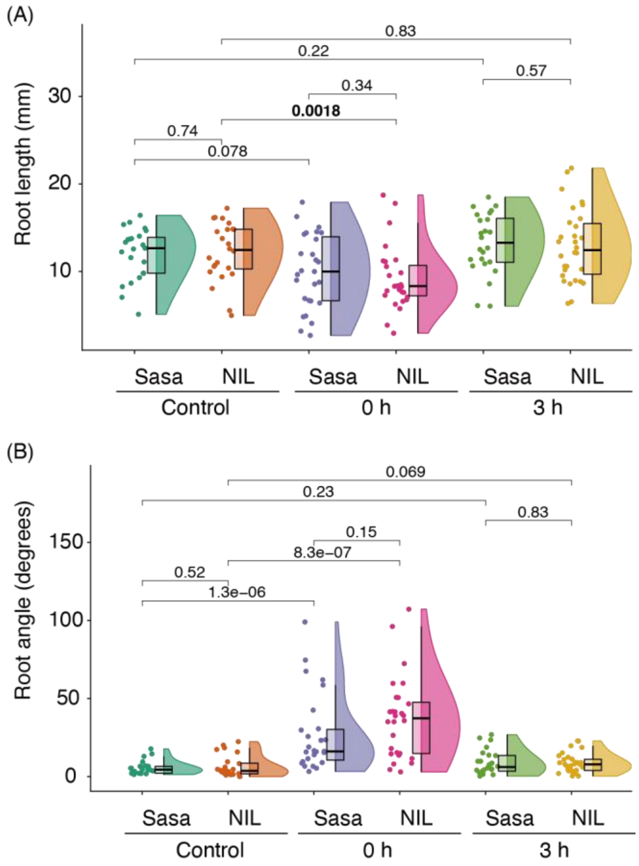

Supplementary Figure 4. Effect of simulated microgravity treatment on roots.

(A) Root elongation (mm). (B) Angle of root elongation relative to the vertical direction. Numerals indicate statistical significance (*p*-values of Wilcoxon rank sum test). Sasa: Sasanishiki, NIL: qsor1-NIL.

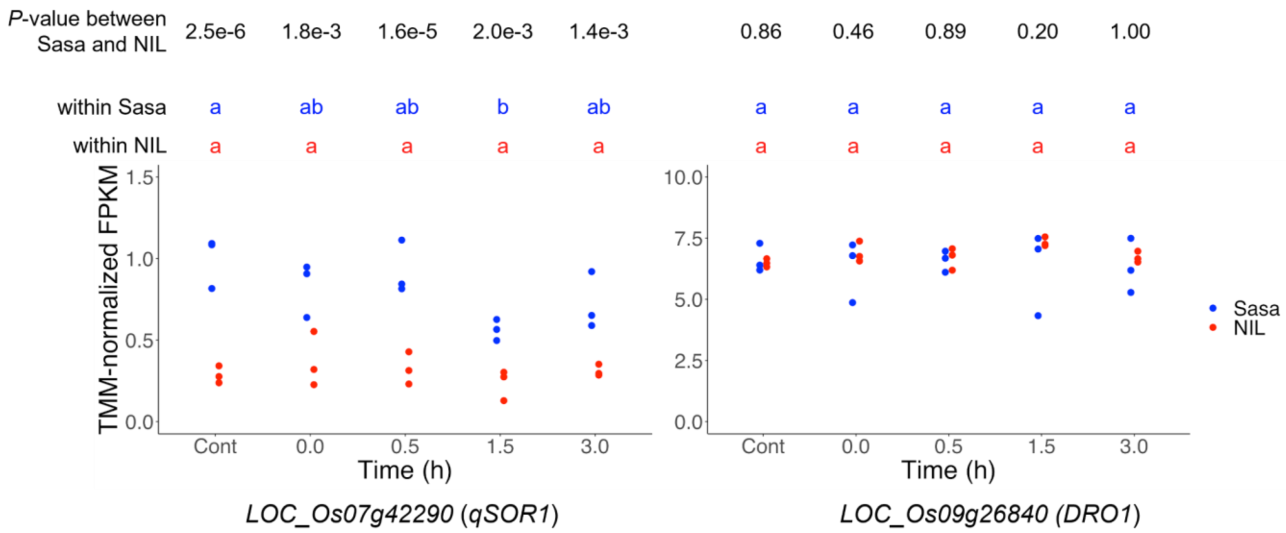

Supplementary Figure 5. Expression levels of *DRO1* and *qSOR1*.

TMM-normalized FPKM values for *qSOR1* (left) and *DRO1* (right). *P*-values between Sasanishiki and qsor1-NIL at each time point are shown at the top. Values labeled with different letters differ significantly among the five time points for Sasa (blue) and NIL (red), respectively ($p < 0.05$, Tukey's HSD test). Sasa: Sasanishiki, NIL: qsor1-NIL.

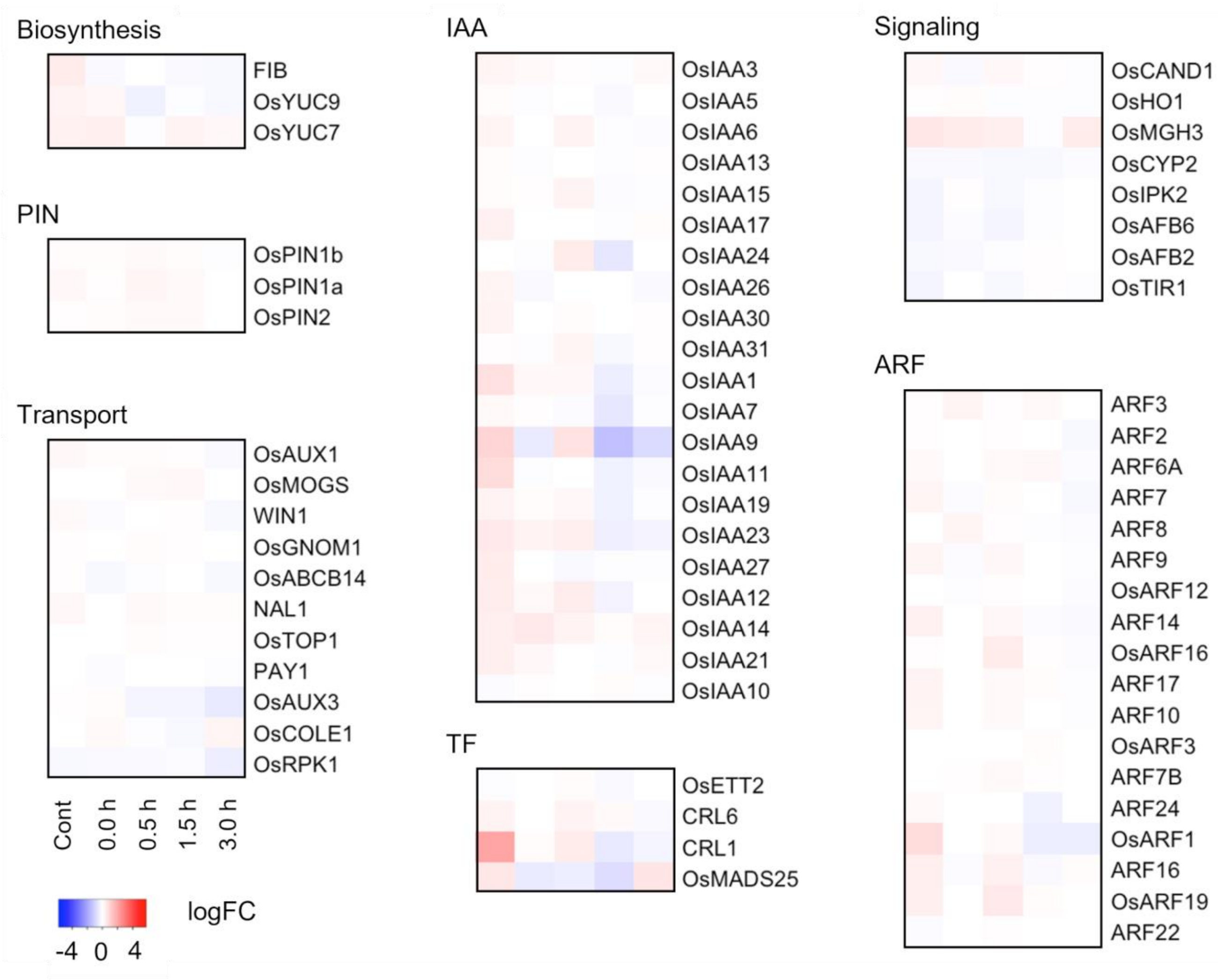

Supplementary Figure 6. Comparison of the expression of auxin-related genes between Sasanishiki and qsor1-NIL at each time point.

Biosynthesis: Auxin biosynthesis genes, PIN: Auxin efflux carrier PIN, Transport: Auxin transport-related genes, IAA: early auxin-responsive genes of auxin/indoleacetic acid, TF: Transcription factor, Signaling: Auxin signaling-related genes, ARF: Auxin response factors.

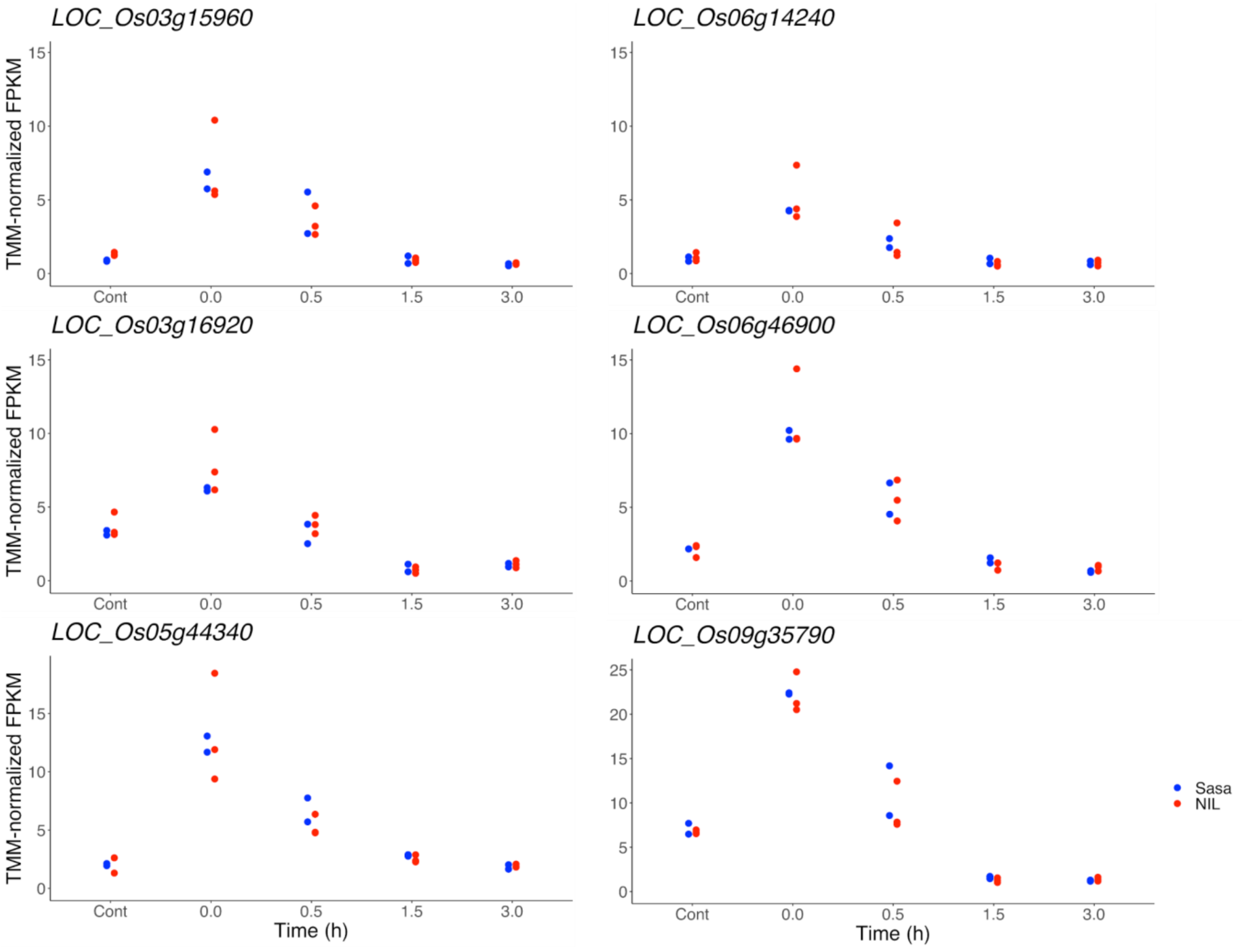

Supplementary Figure 7. Expression levels of six genes of the 'response to heat' and 'response to abiotic stimulus' GO terms enriched in down-regulated DEGs at 0.5 h as in qsor1-NIL (Fig. 3).

Sasa: Sasanishiki. NIL: qsor1-NIL.

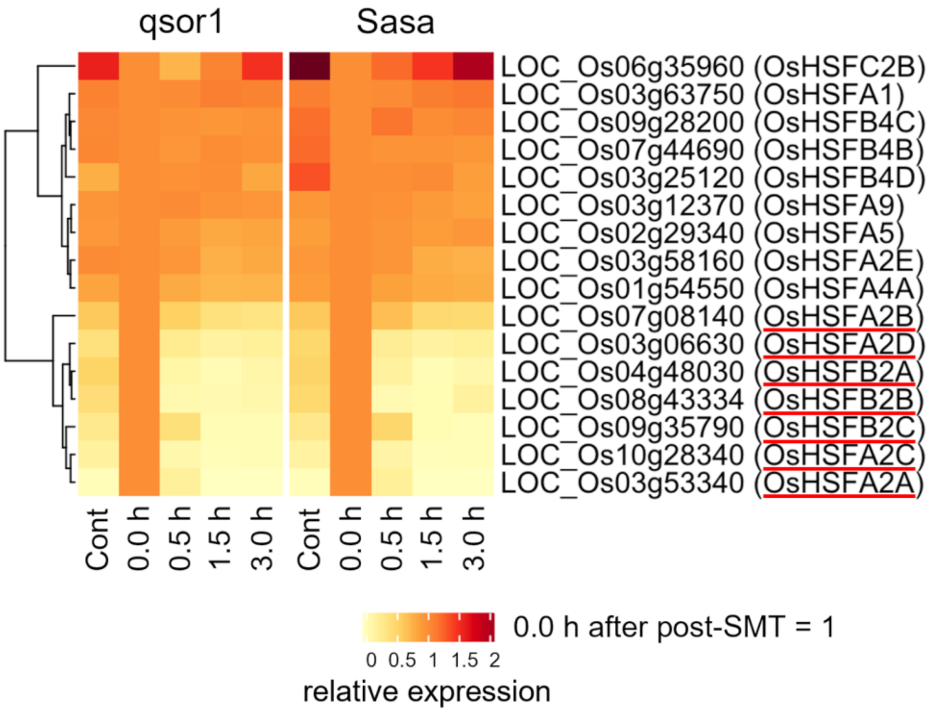

Supplementary Figure 8. Change in relative gene expression of *HSF* when the expression level of 0 h was set to 1 of relative expression.

The 16 *OsHSF* genes whose expression was detected by this RNA-seq are shown. The seven genes detected as down-regulated DEGs during fGS are underlined in red.

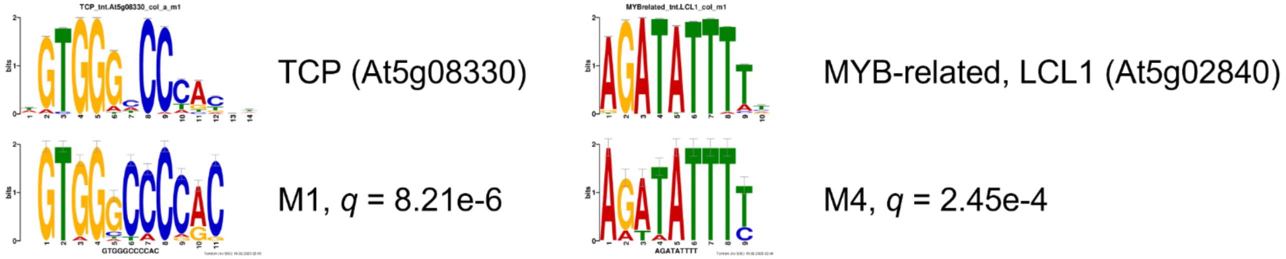

Supplementary Figure 9. Representative motifs found in the 200-bp upstream sequences of the M1 and M4 genes (bottom; MEME program) and corresponding DAP-seq results (top; TOMTOM program; O'Malley et al., 2016).

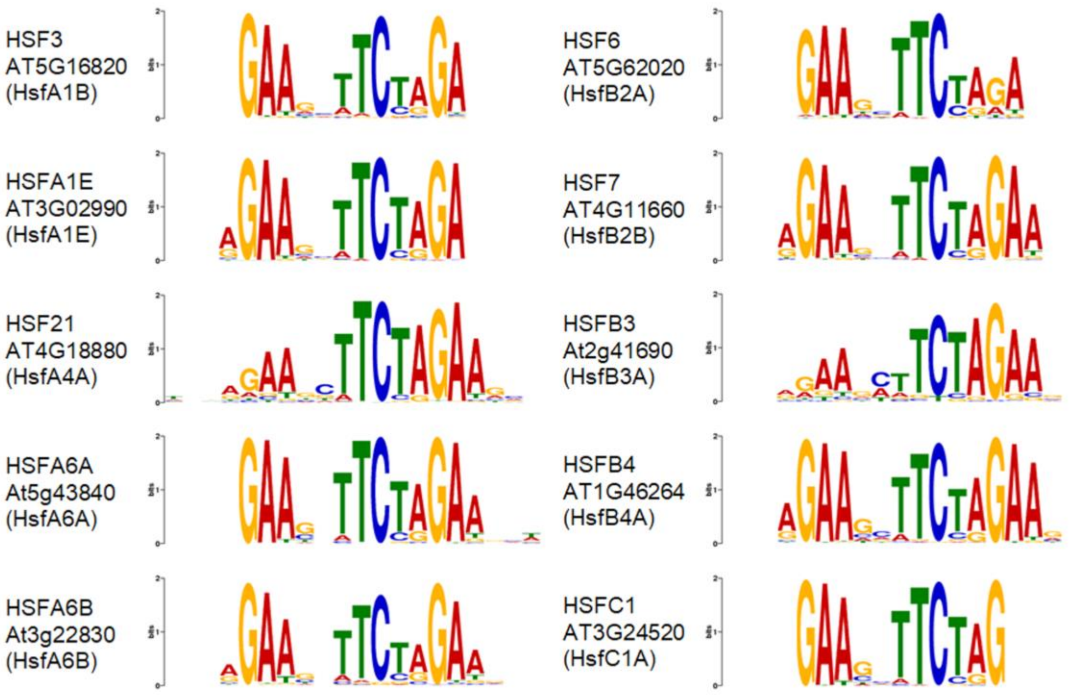

Supplementary Figure 10. DAP-seq motifs of the *Arabidopsis HSF*s (O'Malley et al. 2016).

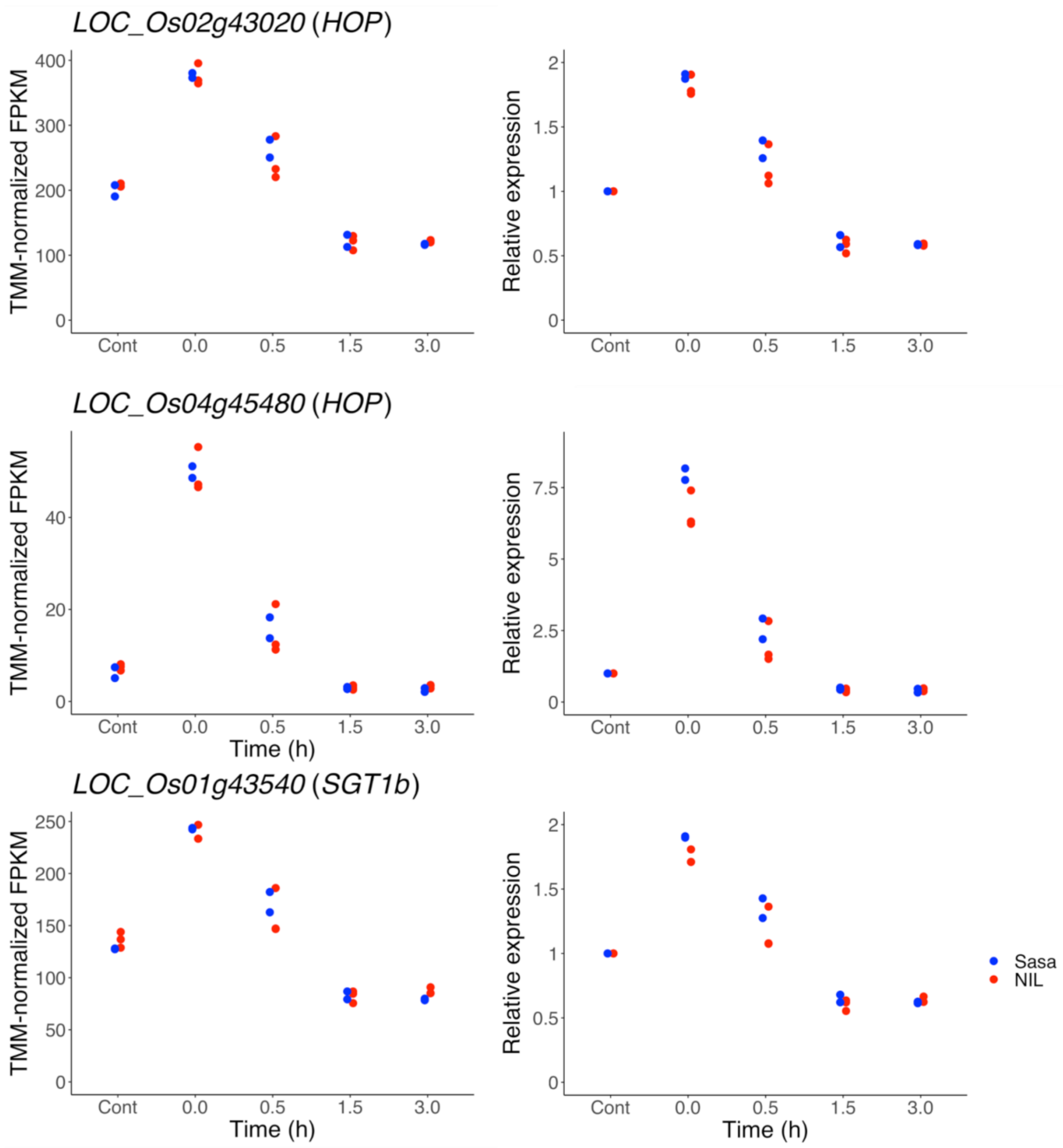

Supplementary Figure 11. Genes for the *HSP90* co-chaperone, *HOP*s and *SGT1*, detected as down-regulated DEGs after fGS.

TMM-normalized FPKM values (left) and expression levels relative to the control without SMT (right) for the rice homologs of *AtHOP*s and *AtSGT1b*. Sasa: Sasanishiki, NIL: qsor1-NIL.

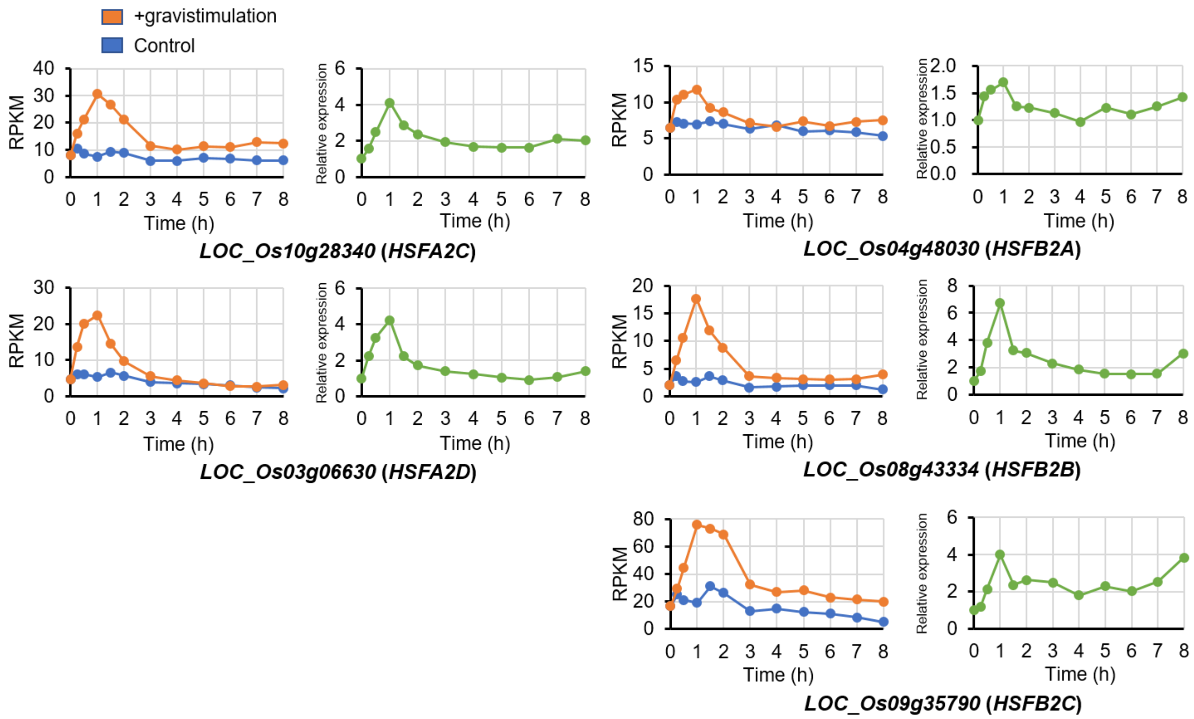

Supplementary Figure 12. Expression of the five HSFs in the gravity-stimulated rice shoot (Zhang et al., 2018).

The figures clearly show that *HSFA2C, HSFA2D*, *HSFB2A*, *HSFB2B*, and *HSFB2C* are up-regulated in response to gravity. The expression of *HSFA2A* and *HSFA2B* among 7 genes in Fig. 5 were below the detection limit of RNA-seq in the rice shoots.

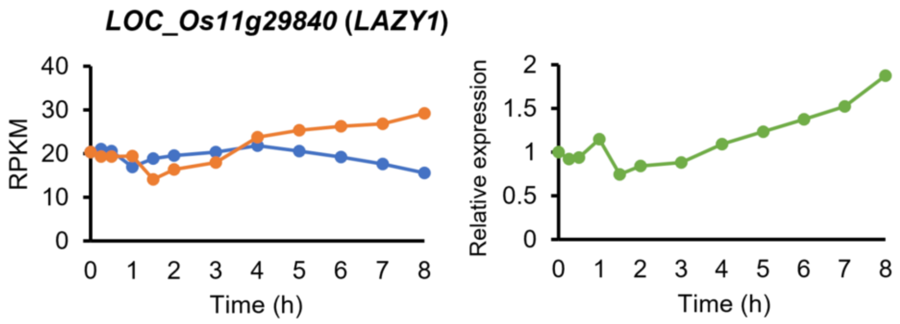

Supplementary Figure 13. Expression of *LAZY1* in the gravity-stimulated rice shoot (Zhang et al., 2018).
